# A Study of Visual Outcomes and Spectral Domain Optical Coherence Tomography (SD-OCT) Biomarker Changes in Patients Treated With Ranibizumab for Diabetic Macular Edema in a Tertiary Hospital

**DOI:** 10.7759/cureus.82520

**Published:** 2025-04-18

**Authors:** Rashmitha Somagani, Manpreet Malik, Rohan Somagani, Kuruva Rakesh Kumar

**Affiliations:** 1 Department of Ophthalmology, Employee's State Insurance Corporation (ESIC) Medical College and Hospital, Hyderabad, IND; 2 Department of Radiology, Nova Diagnostic Centre, Hyderabad, IND

**Keywords:** diabetic macular edema, ranibizumab, retinal biomarkers, spectral-domain oct, visual acuity

## Abstract

Introduction: Diabetic macular edema (DME) is a leading cause of vision impairment in diabetes mellitus. Spectral-domain optical coherence tomography (SD-OCT) provides valuable biomarkers for assessing disease severity and treatment response. This study evaluates the visual outcomes and SD-OCT biomarker changes in patients treated with intravitreal ranibizumab at a tertiary hospital.

Materials and methods: A prospective cohort study was conducted on 50 Type 2 diabetes mellitus patients with center-involving DME at Employees' State Insurance Corporation (ESIC) Medical College and Hospital, Hyderabad, from July 2022 to December 2023. Inclusion criteria were best-corrected visual acuity (BCVA) <6/9 and central retinal thickness (CRT) ≥280 µm. Exclusion criteria included other retinal diseases, vision-impairing cataracts, or glaucoma. Patients received three monthly intravitreal ranibizumab injections and were followed up at one, two, three, and six months. BCVA, CRT, and SD-OCT biomarkers such as hyperreflective foci (HRF), subretinal neuroretinal detachment (SND), intraretinal cyst (IRC) size, disorganization of retinal inner layers (DRIL), ellipsoid zone (EZ) disruption, and external limiting membrane (ELM) disruption were assessed. Statistical analysis was performed using SPSS software, version 26 (IBM Corp., Armonk, NY).

Results: BCVA improved from 0.76±0.39 at baseline to 0.37±0.29 at six months (p<0.00001). CRT reduced from 473.66±111.65 µm to 326.64±71.37 µm (p<0.00001). HRF, SND, and IRC size showed significant regression. DRIL, EZ, and ELM disruption improved significantly (p<0.00001). There was no statistically significant association between OCT biomarker features and BCVA improvement of more than three lines.

Conclusion: Intravitreal ranibizumab significantly improves visual acuity and reduces retinal thickness in DME patients. SD-OCT biomarkers are valuable in monitoring treatment response and disease progression.

## Introduction

Diabetic macular edema (DME) is a leading cause of vision impairment in patients with diabetes mellitus, characterized by the accumulation of extracellular fluid in the macula due to increased vascular permeability [1]. It commonly affects the inner nuclear layer, outer plexiform layer, Henle’s fiber layer, and subretinal space. DME is a manifestation of diabetic retinopathy (DR), a microvascular complication resulting from prolonged hyperglycemia, leading to retinal damage [2,3]. Risk factors for DME and DR include poor glycaemic control, long duration of diabetes, hypertension, hyperlipidemia, renal dysfunction, and the use of thiazolidinediones. Secondary risk factors such as elevated HbA1c levels further contribute to disease progression [4,5].

The prevalence of DME varies globally and regionally. In India, the prevalence of diabetic retinopathy ranges from 17.6% to 28.2%, with sight-threatening DR, including DME and proliferative DR, affecting less than 10% of diabetics [6]. In urban South India, the prevalence of DME is estimated at 2.1%, with center-involving DME at 3.03% and non-center-involving DME at 10.8% [7]. On a global scale, the estimated number of DME cases is around 19 million, projected to increase to 29 million by 2045, highlighting the growing burden of this condition [8]. Given the significant impact of DME on vision and quality of life, effective diagnostic and therapeutic strategies are essential.

Spectral-domain optical coherence tomography (SD-OCT) has revolutionized the diagnosis and monitoring of DME by providing high-resolution cross-sectional images of the retina. SD-OCT biomarkers such as central retinal thickness (CRT), disorganization of retinal inner layers (DRIL), and hyperreflective foci (HRF) serve as crucial indicators of disease severity and treatment response [9]. Intravitreal anti-vascular endothelial growth factor (VEGF) agents, particularly ranibizumab, have become the cornerstone of DME treatment, demonstrating significant improvements in visual acuity in multiple clinical trials [10]. However, there remains a need to further evaluate the correlation between SD-OCT biomarkers and visual outcomes in real-world clinical settings, particularly in tertiary care hospitals, to optimize patient management strategies. This study aims to assess the changes in visual acuity and SD-OCT biomarkers following intravitreal ranibizumab therapy in patients with center-involving diabetic macular edema (DME), and to explore potential correlations between these biomarkers and visual improvement.

## Materials and methods

**Figure 1:**
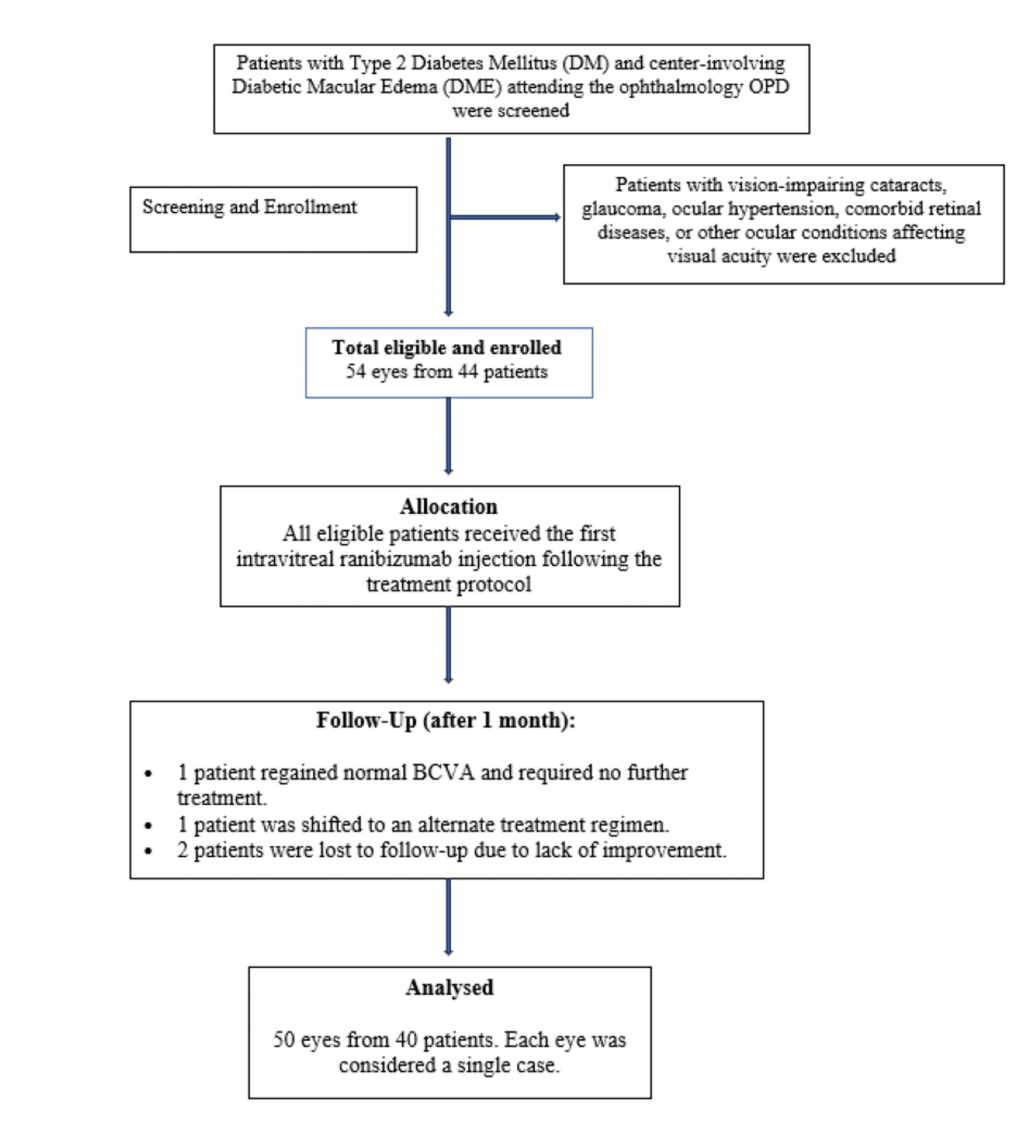
Consort flow diagram

This prospective cohort study was conducted on Type 2 diabetes mellitus (DM) patients with center-involving DME attending the ophthalmology outpatient department of the Employees' State Insurance Corporation (ESIC) Medical College and Hospital over a one-year period from July 2022 to December 2023. Patients were included in the study if they had DME with a best-corrected visual acuity (BCVA) of less than 6/9 and a CRT of ≥280 µm on SD-OCT. Patients who did not provide informed consent and those with comorbid retinal diseases, vision-impairing cataracts, glaucoma, ocular hypertension, iris neovascularization, or other ocular conditions affecting visual acuity were excluded (Figure 1).

An approval for this study was obtained from the Institutional Ethics Committee of the Employees' State Insurance Corporation (ESIC) Medical College and Hospital before initiating the study. Written informed consent was obtained from all participants. A detailed ocular and systemic history was recorded, followed by a comprehensive ocular examination. BCVA was measured using a Snellen’s visual acuity chart, and slit-lamp biomicroscopy was performed. Fundus examination was conducted using a +90D lens under a slit-lamp biomicroscope and a +20D lens with an indirect ophthalmoscope. Intraocular pressure (IOP) was measured using non-contact tonometry (NCT). Fundus photography (color and red-free) was performed using a Zeiss fundus photography machine, and SD-OCT assessments of CRT, sensory neural detachment (SND), hyperreflective retinal spots (HRS), intra-retinal cyst (IRC) size, disorganization of inner retinal layers (DRIL), ellipsoid zone (EZ) disruption, and external limiting membrane (ELM) disruption were carried out at every visit.

CRT was defined as the thickness of the central 1-mm diameter circle on the ETDRS® (Early Treatment of Diabetic Retinopathy Study) grid map. SND appeared on OCT as a hypo-reflective area beneath the neuroretina. HRF were identified as hyperreflective spots >30 µm with back shadowing, located in the outer retina, with reflectivity similar to the retinal pigment epithelium (RPE)-Bruch’s membrane complex. DRIL was characterized by the inability to delineate the boundaries of the ganglion cell-inner plexiform layer (GCL-IPL) complex, inner nuclear layer (INL), and outer plexiform layer (OPL). The EZ, also known as the inner segment-outer segment (IS-OS) junction, was visualized as a hyperreflective line just above the photoreceptors.

All patients received three monthly intravitreal ranibizumab injections as a loading dose, with follow-ups conducted after each injection and an additional follow-up at six months. The treatment protocol followed the Diabetic Retinopathy Clinical Research Network guidelines for DME with diabetic retinopathy (DR). Intravitreal injections were administered under aseptic conditions. After topical anesthesia, 0.3 mg (0.05 mL) of anti-VEGF ranibizumab was injected into the inferotemporal quadrant of the eyeball. The injection was placed 3.5 mm from the limbus in pseudophakic eyes and 4 mm from the limbus in phakic eyes using a 30-gauge needle.

All statistical analyses were performed using SPSS software, version 26 (IBM Corp., Armonk, NY). Continuous variables were expressed as mean ± standard deviation (SD), while categorical variables were presented as frequencies and percentages. A one-way ANOVA test was used to analyze changes in visual acuity, CRT, and OCT biomarkers over time. A p-value of <0.05 was considered statistically significant.

## Results

A total of 54 eyes from 44 patients who met the inclusion and exclusion criteria were included in the study. One patient regained normal BCVA after the second injection, one patient was shifted to an alternate treatment regimen, and two patients were lost to follow-up after the second injection due to lack of improvement. Ultimately, 50 eyes from 40 patients were analyzed, with each eye considered as a single case.

The study included 50 patients diagnosed with DME and treated with intravitreal ranibizumab. The majority of the participants were between 51 and 60 years old (48%), followed by those over 60 years (28%). Males comprised 70% of the study population, while females accounted for 30%. The laterality of eye involvement was nearly equal, with 52% of cases affecting the right eye and 48% affecting the left eye (Table 1).

**Table 1:** Demographic and clinical characteristics of the study population Frequency and percentages were calculated. OD: Oculus dexter, OS: Oculus sinister.

Variable		Frequency (%)
Age	< 40 years	2 (5%)
	41-50 years	8 (20%)
	51-60 years	19 (48%)
	> 60 years	11 (28%)
Gender	Male	28 (70%)
	Female	12 (30%)
Eye Distribution	OD (Right)	26 (52%)
	OS (Left)	24 (48%)

The severity of diabetic retinopathy among the participants varied, with early proliferative diabetic retinopathy (PDR) with DME being the most common diagnosis (30%). Moderate non-proliferative diabetic retinopathy (NPDR) with DME was observed in 20% of cases, while severe and very severe NPDR with DME were present in 10% of cases each. A smaller proportion of patients (4%) had late PDR with DME, and 16% had mild NPDR with DME without additional complications. These findings highlight the diverse severity spectrum of diabetic retinopathy in patients undergoing treatment for DME (Table 2).

**Table 2:** Distribution of diabetic retinopathy severity with DME Frequency and percentages were calculated. NDPR: Non-proliferative diabetic retinopathy; DME: Diabetic macular edema, PDR: Proliferative diabetic retinopathy.

Diagnosis	Frequency (%)
Mild NPDR with DME	8 (16%)
Mild NPDR with DME + ERM	2 (4%)
Mild to Moderate NPDR with DME	3 (6%)
Moderate NPDR with DME	10 (20%)
Severe NPDR with DME	5 (10%)
Very Severe NPDR with DME	5 (10%)
Early PDR with DME	15 (30%)
Late PDR with DME	2 (4%)
Total	50 (100%)

Visual acuity and central retinal thickness (CRT) showed significant improvement over the six-month follow-up period. Baseline visual acuity was 0.76 ± 0.39, which improved progressively to 0.37 ± 0.29 at six months (p < 0.00001). Similarly, CRT reduced from a baseline value of 473.66 ± 111.65 µm to 326.64 ± 71.37 µm at six months (p < 0.00001). Notably, 68% of patients demonstrated a visual acuity improvement of more than three lines, and 88% exhibited a CRT reduction greater than 100 µm, indicating a positive therapeutic response to ranibizumab (Table 3).

**Table 3:** Changes in visual acuity and central retinal thickness over six months Mean and SD were calculated at each follow-up. One way ANOVA was applied and a p-value less than 0.05 was considered significant. VA: Visual acuity, CRT: Central retinal thickness.

Follow-up Time	Visual Acuity (Mean ± SD)	Central Retinal Thickness (Mean ± SD)
Baseline	0.76 ± 0.39	473.66 ± 111.65 μm
1 Month	0.63 ± 0.37	415.74 ± 104.0 μm
2 Months	0.50 ± 0.34	363.08 ± 80.9 μm
3 Months	0.37 ± 0.30	328.67 ± 72.28 μm
6 Months	0.37 ± 0.29	326.64 ± 71.37 μm
f-value	12.7136	22.8763
p-value	< 0.00001	< 0.00001
Cases with improvement	34 (68%): VA >3-line improvement	44 (88%): CRT >100μm reduction

OCT biomarkers such as hyperreflective foci (HRF), subretinal neuroretinal detachment (SND), and intraretinal cyst (IRC) size demonstrated significant regression following treatment. HRF decreased from 11.38 ± 11.6 at baseline to 5.40 ± 8.09 at six months (p = 0.0015). SND reduced significantly from 66.86 ± 98.0 to 15.74 ± 35.14 (p = 0.0001), and IRC size decreased from 890.54 ± 378.91 to 224.51 ± 285.20 (p < 0.00001). Among these biomarkers, SND showed the highest rate of improvement, with 82% of cases exhibiting resolution by six months (Table 4).

**Table 4:** Changes in OCT biomarkers over six months Mean and SD were calculated at each follow-up. One way ANOVA was applied and a p-value less than 0.05 was considered significant. OCT: Optical coherence tomography, HRF: Hyperreflective foci, SND: Subretinal neuroretinal detachment, IRC: Intraretinal cyst.

Follow-up Time	HRF (Mean ± SD)	SND (Mean ± SD)	IRC Size (Mean ± SD)
Baseline	11.38 ± 11.6	66.86 ± 98.0	890.54 ± 378.91
One Month	12.80 ± 14.9	48.46 ± 66.64	612.60 ± 364.02
Two Months	8.22 ± 10.4	31.78 ± 51.19	363.58 ± 344.12
Three Months	5.68 ± 8.27	16.09 ± 35.45	238.68 ± 289.31
Six Months	5.40 ± 8.09	15.74 ± 35.14	224.51 ± 285.20
f-value	4.6038	5.6252	35.8208
p-value	0.0015	0.0001	< 0.00001
Cases with improvement	30 (60%)	41 (82%)	22 (44%)

The structural integrity of retinal layers improved over the course of treatment. The presence of disorganization of retinal inner layers (DRIL) declined from 60% at baseline to 12% at six months (p < 0.00001). Similarly, disruption of the ellipsoid zone (EZ) and external limiting membrane (ELM) showed a significant reduction from 64% and 60% at baseline to 10% at six months, respectively (p < 0.00001). The progressive restoration of retinal microstructures further corroborates the beneficial effects of ranibizumab in diabetic macular edema (Table 5).

**Table 5:** Structural changes in retinal layers over six months Frequency and percentages were calculated for each variable. Chi-square test was applied and a p-value less than 0.05 was considered significant. DRIL: Disorganization of retinal inner layers, EZ: Ellipsoid zone, ELM: External limiting membrane.

Follow-up Time	DRIL Present (%)	EZ Disrupted (%)	ELM Disrupted (%)
Baseline	30 (60%)	32 (64%)	30 (60%)
One Month	25 (50%)	24 (48%)	23 (46%)
Two Months	19 (38%)	20 (40%)	20 (40%)
Three Months	8 (16%)	8 (16%)	8 (16%)
Six Months	6 (12%)	5 (10%)	5 (10%)
Chi-square value	35.1714	40.6998	35.7625
p-value	< 0.00001	< 0.00001	< 0.00001

Further analysis was conducted by dividing the study population into two subgroups based on visual acuity improvement after six months of ranibizumab treatment: Subgroup A (≥3 lines improvement in BCVA, n = 34) and Subgroup B (<3 lines improvement in BCVA, n = 16). The association between OCT biomarker features and visual outcomes was evaluated by comparing the presence or absence of each feature across the two subgroups. CRT reduction of less than 100 µm was observed in 11.8% of Subgroup A and 12.5% of Subgroup B, showing no significant difference (p = 0.943). Hyperreflective foci were more frequently observed in Subgroup A (41.2%) compared to Subgroup B (37.5%), but the difference was not statistically significant (p = 0.805). Similarly, other biomarkers such as sensory neural detachment (p = 0.919), intraretinal cyst size (p = 0.233), disorganization of retinal inner layers (DRIL) (p = 0.295), ellipsoid zone (EZ) disruption (p = 0.697), and external limiting membrane (ELM) disruption (p = 0.697) did not show significant associations with visual acuity improvement. These findings suggest that although structural changes were observed over time, the presence or absence of individual OCT biomarker features at six months was not significantly associated with the degree of visual improvement (Table 6).

**Table 6:** Analysis of OCT biomarker features in subgroups A and B at six months Frequency and percentages were calculated for all varibles. Chi-square test was applied and a p-value less than 0.05 was considered significant. CRT: Central retinal thickness, ELM: External limiting membrane.

OCT Biomarker Feature	Subgroup A (BCVA > 3 lines improvement) (n = 34)		Subgroup B (BCVA < 3 lines improvement) (n = 16)		Chi-square value	p-value
	Present	Absent	Present	Absent		
CRT (<100µm) Reduction	4 (11.8%)	30 (88.2%)	2 (12.5%)	14 (87.5%)	0.0055	0.943
Hyperreflective Foci	14 (41.2%)	20 (58.8%)	6 (37.5%)	10 (62.5%)	0.0612	0.805
Sensory Neural Detachment	6 (17.6%)	28 (82.4%)	3 (18.8%)	13 (81.2%)	0.0089	0.919
Intraretinal Cyst Size	21 (61.8%)	13 (38.2%)	7 (43.8%)	9 (56.2%)	1.4329	0.233
Disorganization of Retinal Inner Layers	3 (8.8%)	31 (91.2%)	3 (18.8%)	13 (81.2%)	1.0152	0.295
Ellipsoid Zone Disruption	3 (8.8%)	31 (91.2%)	2 (12.5%)	14 (87.5%)	0.1633	0.697
ELM Disruption	3 (8.8%)	31 (91.2%)	2 (12.5%)	14 (87.5%)	0.1633	0.697

## Discussion

Anti-vascular endothelial growth factor (VEGF) therapy has emerged as the first-line treatment for DME, with intravitreal ranibizumab demonstrating significant efficacy. The present study aimed to analyze the effects of intravitreal ranibizumab treatment on visual outcomes and SD-OCT biomarkers in patients with DME. Our study included 40 patients, with a mean age of 55.82 years, which is comparable to the study by Raman et al., where the mean age was 56.32 ± 10.02 years [5]. The gender distribution in our study consisted of 36 males and 14 females, while Raman et al. reported a more balanced distribution of 750 males and 664 females [5]. Similarly, Chang et al. included 22 males and 13 females in their study [11]. The disease severity distribution in our study showed that DME was most prevalent in early PDR (30%), followed by moderate NPDR (20%) and mild NPDR with DME (8%). In contrast, Chang et al. reported a higher prevalence of PDR (64.6%) compared to severe NPDR (1.5%), with 33.9% of cases receiving prior pan-retinal photocoagulation (PRP) treatment [11].

Visual acuity (VA) significantly improved following intravitreal ranibizumab treatment. Our study observed a mean increase in logMAR VA of 0.39 at the end of six months, with baseline VA improving from 0.76 to 0.63, 0.50, and 0.37 in subsequent follow-ups. A statistically significant mean improvement of 0.39 was noted up to the third follow-up (p-value < 0.00001), after which VA stabilized up to six months. Mahapatra et al. [12] reported a mean logMAR VA improvement of 0.55 at six months, while Chang et al. [11] documented a mean VA improvement of 0.42 at six months and 0.36 at 12 months. Our findings align with previous studies, supporting the efficacy of ranibizumab in improving visual outcomes in DME patients.

A significant reduction in central retinal thickness (CRT) was observed in our study population, with mean CRT decreasing from 473.66 ± 111.65 µm at baseline to 326.64 ± 71.37 µm at six months. This finding is comparable to the study by Mahapatra et al., where the mean CRT reduced from 405 µm at baseline to 278.35 µm at six months [12]. Additionally, our study demonstrated that patients with a CRT reduction greater than 100 µm had better visual outcomes at six months compared to those with a reduction of less than 100 µm. These findings support the predictive value of CRT reduction in assessing visual prognosis, consistent with the results reported by Chang et al. [11].

We also observed a significant reduction in SD-OCT biomarkers, including hyperreflective foci (HRF) (72% to 40%), sensory neural detachment (SND) (44% to 18%), intraretinal cyst (IRC) size (98% to 56%), disorganization of inner retinal layers (DRIL) (60% to 12%), ellipsoid zone (EZ) disruption (64% to 10%), and external limiting membrane (ELM) disruption (60% to 10%). Similar reductions in IRC (64.6% to 49.2%) and HRF following IVR treatment (p < 0.05) were reported by Chang et al. [11].

Subgroup analysis revealed that OCT biomarkers such as SND, DRIL, EZ, and ELM disruption showed greater reduction in subgroup A than subgroup B, making them potential prognostic indicators. SND showed up to 75% improvement in subgroup A but less than 15% in subgroup B, supporting its role as a predictor of visual outcomes. DRIL, EZ, and ELM disruption demonstrated up to 99% reduction in subgroup A and 45% in subgroup B, further emphasizing their prognostic significance. HRF reduction was similar in both subgroups (50%), while IRC size showed greater reduction in subgroup B (55%) than in subgroup A (35%). Sun et al. reported that the absence of DRIL at baseline and a decrease in its extent during treatment correlated with better visual outcomes [13]. However, Radhakrishnan et al. concluded that DRIL was not a reliable predictor of visual outcomes [14]. Zur et al. identified the absence of HRF and an intact EZ as good predictors of visual prognosis [15].

Our findings suggest that OCT biomarkers such as SND, DRIL, EZ, and ELM disruption are strong prognostic indicators in DME, whereas HRF and IRC size do not have significant predictive value. However, further large-scale, statistically robust studies are required to validate the prognostic significance of these biomarkers and improve the classification and treatment stratification of DME based on SD-OCT findings.

## Conclusions

Our study underscores the clinical efficacy of intravitreal ranibizumab in the management of diabetic macular edema (DME), demonstrating significant improvements in visual acuity and a marked reduction in central retinal thickness over a six-month follow-up period. Importantly, optical coherence tomography (OCT) biomarkers played a critical role in predicting treatment response and long-term visual outcomes. Specifically, the presence of subfoveal neurosensory detachment (SND), disorganization of the retinal inner layers (DRIL), and disruption of the ellipsoid zone (EZ) and external limiting membrane (ELM) were identified as strong prognostic indicators of poor visual recovery. In contrast, hyperreflective foci (HRF) and intraretinal cyst (IRC) size exhibited limited prognostic significance. Notably, a reduction in central retinal thickness exceeding 100 µm following treatment was significantly associated with better visual improvement at the six-month mark. These findings highlight the utility of OCT biomarkers not only in monitoring anatomical response but also in predicting functional outcomes, thereby reinforcing their value in the personalized management of DME patients undergoing anti-VEGF therapy with ranibizumab.
